# Pioneering Informed Consent for Return of Research Results to Breast Cancer Patients Facing Barriers to Implementation of Genomic Medicine: The Kenyan BRCA1/2 Testing Experience Using Whole Exome Sequencing

**DOI:** 10.3389/fgene.2020.00170

**Published:** 2020-03-06

**Authors:** Rispah Torrorey-Sawe, Nicole van der Merwe, Simeon Kipkoech Mining, Maritha J. Kotze

**Affiliations:** ^1^Division of Chemical Pathology, Department of Pathology, Faculty of Medicine and Health Sciences, Stellenbosch University, Tygerberg, South Africa; ^2^Department of Immunology, School of Medicine, College of Health Sciences, Moi University, Eldoret, Kenya; ^3^National Health Laboratory Service, Tygerberg Hospital, Cape Town, South Africa

**Keywords:** informed consent, genetics, genomics, pathology, return of results, Africa

## Abstract

**Introduction:**

Obtaining informed consent from study participants and disseminating the findings responsibly is a key principle required for ethically conducted clinical and genetic research. Reports from African researchers providing feedback on insights gained during the return of whole exome sequencing (WES) results to breast cancer patients treated in resource-limited settings is lacking.

**Aim:**

The empirical process used to fill this gap in relation to *BRCA1/2* variant detection using WES provided unique insights incorporated into a pathology-supported genetic testing algorithm for return of research results to Kenyan breast cancer patients.

**Methods:**

The Informed consent form approved by the Moi Teaching and Referral Hospital in Kenya was adopted from a translational research study conducted in South Africa. Initially, the informed consent process was piloted in 16 Kenyan female patients referred for breast surgery, following a community-based awareness campaign. A total of 95 female and two male breast cancer patients were enrolled in the study from 2013 to 2016. Immunohistochemistry (IHC) results of estrogen receptor (ER), progesterone receptor (PR) and human epidermal growth factor receptor-2 (HER2) status were obtained from hospital records. DNA of patients with a family history of cancer was extracted from saliva and screened for pathogenic variants in the *BRCA1/2* genes as the first step using WES.

**Results:**

Ten patients approached for participation in this study declined to sign the informed consent form. Data on IHC used as a proxy for molecular subtype were available in 8 of 13 breast cancer patients (62%) with a family history of cancer. Five *BRCA*1/2 variants of uncertain clinical significance were detected, as well as a pathogenic *BRCA2* variant (c.5159C > A; S1720^∗^) in a female patient eligible for return of WES results.

**Conclusion:**

Experience gained during the qualitative pilot phase was essential to overcome challenges associated with the translation of sophisticated genetic terms into native African languages. Detection of a pathogenic *BRCA*2 variant in a patient with familial breast cancer, frequently associated with hormone receptor-positive breast carcinoma as reported in this case, led to a high level of confidence on which to base risk management in future. Implementation of new technologies alongside standard pathology provides a practical approach to the application of genomic medicine in Africa.

## Introduction

A gap in knowledge exists regarding the application of national and international ethical guidelines for research in Africa and other resource-poor settings (Alfano, 2013). Language and cultural barriers complicate the process of obtaining informed consent for genetic studies in rural African settings and data on solutions based on real-life experience are limited (Bhutta, 2004; de Vries et al., 2015; Adebamowo et al., 2018). In Kenya, obtaining informed consent for research and return of genetic results is a major challenge as the consent process is required to meet international standards, despite cultural diversity and lack of genetic counsellors or other healthcare professionals with experience in genomic research.

No standard format exists for obtaining informed consent from research participants applicable to both developed and developing countries. Careful planning is therefore required to ensure that ethical values are applied appropriately, especially in African countries where genomic research has not previously been performed (Afolabi et al., 2014; de Vries et al., 2015). Most emphasis appears to be placed on participant enrollment rather than ensuring a thorough understanding and comprehension of the project goals (Bhutta, 2004; Marshall, 2007; Dekking et al., 2014). A successful consenting process requires that potential study participants are brought to a high level of understanding how the purpose, methods, and risks of a study related to their personal or family medical conditions (Emanuel, 2000; Gupta et al., 2012). Good ethical conduct requires that the participant is allowed to make a voluntary and uncoerced decision whether or not to participate in a given study (Gupta et al., 2012). Therefore, only by obtaining true informed consent before the commencement of a study, can the standards for respect for people be achieved (Munung et al., 2016). Previous studies have shown that consenting illiterate individuals may be intimidating to them, which can further exacerbate challenges experienced during the consenting process (Alaei et al., 2013; Marshall et al., 2014). Even among literate patients, some may think that signing a consent form for research is part of their treatment regime (Bhutta, 2004). Therefore, managing participant expectations about feedback of research findings, either in a group context or on an individual basis, is an important part of the informed consent process.

Many approaches for return of research results have been reported. Knoppers et al. (2015) summarized these into four methods considered appropriate in the era of whole exome/genome sequencing. The first involves the use of filters, specific gene panels or targeted sequencing to reduce the potential for variants of uncertain clinical significance (VUS) and incidental findings. In the second approach, research results can only be returned when requirements for analytical validity, clinical significance and actionability (ACA) is met or the findings have personal utility and are clearly of essential relevance to health. The third criterion is a case-by-case approach where the communication of incidental findings obtained both in the research and clinical settings is evaluated on an individual basis. In a research setting, consulting a research ethics committee is required if the feedback plan was not included in the informed consent form (ICF). In the clinical setting, on the other hand, the treating clinician may consult with colleagues and depending on age, prognosis and other personal circumstances of the patient, findings that are outside of the primary indication of the test used in the research may be communicated to the patient (Jarvik et al., 2014). The no-return fourth criterion applies when an individual’s results from genome-based testing within the research context is used for the purpose of producing generalizable findings as opposed to demonstrating clinical utility. Research participants generally express great interest in receiving most classes of genetic findings, especially those with potential clinical significance (Christenhusz et al., 2013; Jarvik et al., 2014). Many research participants reportedly sought all of these results regardless of whether actionable, clinically significant or discovered incidentally (Wolf, 2013; Appelbaum et al., 2014). A multi-disciplinary team of knowledgeable health professionals with experience in genetic testing and interpretation skills are required to provide this kind of information.

Lack of genetic counseling services in Kenya, a country where breast cancer is the most common (∼23%) form of cancer with approximately 75% of patients dying within 5 years of diagnosis (Ministry of Health Kenya, 2013), hampers the incorporation of genomics in clinical practice. Previous studies performed in western Kenya revealed early onset, aggressive breast cancer and lack of routine tumor subtyping to direct optimal treatment (Busakhala and Torrorey, 2012; Sawe et al., 2016, 2017). Development of screening models that can be translated into targeted risk reduction intervention strategies as the purpose of this study, is therefore needed to overcome language and other barriers to personalized genomic medicine in various cultural settings.

According to the Helsinki Declaration, it is an ethical obligation of researchers to make available or publish information about the outcome and results obtained in clinical research (World medical Association, 2013). In African genomic studies, provision for feedback of genetic results to individual study participants is absent in most ICFs used by investigators within the H3Africa (Human Heredity and Health in Africa) Consortium (Munung et al., 2016). de Vries et al. (2017) examined the existing ethics regulatory framework for genomic research and biobanking in 22 African countries including South Africa, and reported that only seven (Botswana, Cameroon, Ethiopia, Rwanda, Malawi, Sudan, and Uganda) specifically refer to the return of genetic results. A general concern is the impact of genetic research on family members, and whether or not these should also be included in the feedback. With regard to incidental research results, disclosure guidelines stipulate that (1) results should not be disclosed to relatives or third parties without written permission (Ethiopia), (2) disclosure should be determined by the investigator based on test sensitivity and specificity (Malawi), (3) participants’ consent is required (Malawi, Botswana, and Cameroon) and mandatory involvement of a genetic counselor (Malawi and Botswana), (4) notification of test availability outside the research setting should carefully consider who has access to study results (Cameroon), and (5) policies for feedback and precautions should be in place to prevent unauthorized disclosure (Sudan). Uganda is the only country that stipulates that any results that are of clinical relevance, including incidental findings, must be returned to study participants. In Kenya no guidelines have been published for return of genomic research results, hence the current study adopted a research translation model developed in South Africa using a pathology-supported genetic testing (PSGT) strategy (Kotze, 2016).

Return of genetic results to study participants is challenged by the complex nature of integrating genetic findings into personal healthcare. A broad range of clinical information needs to be collected for genetic information to be meaningfully interpreted. Furthermore, to prevent unnecessary interventions, development and maintenance of secure open-access database resources are required to perform follow-up surveys and track long-term health outcomes of individual patients. For this purpose, substantial investment has been made in South Africa to develop the PSGT platform at the interface between the research laboratory and clinical practice. In the case of breast cancer, both germline and tumor genetics are taken into account to help guide treatment decisions across the continuum of care (Grant et al., 2013, 2019; Kotze et al., 2015; van der Merwe et al., 2017). The PSGT algorithm developed in South Africa was pioneered in Kenya to return actionable research results to patients with breast cancer. The ethical framework developed for this purpose provides a sound basis for clinical intervention during and beyond the course of a single research objective (Baatjes, 2018). The problems encountered and insights gained using an informed consent document that made provision for the return of genomic results, helped to inform the implementation of PSGT in Kenya on an individual basis.

## Materials and Methods

### Ethics Approval

The Moi University-MTRH Institutional Ethics and Research Committee approved the study under project number 000655. Renewal of this project has been approved on an annual basis toward the return of research results from 2019.

### Study Design

**FIGURE 1:**
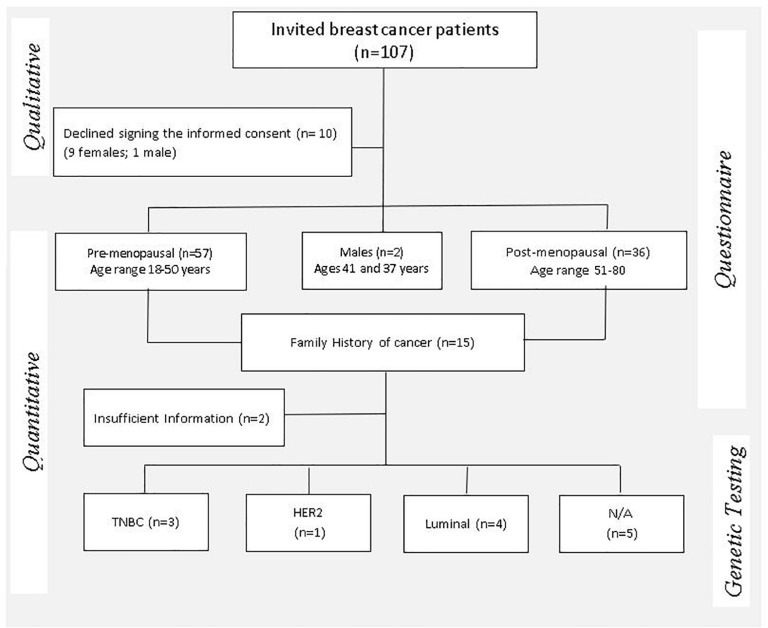
Mixed methods study design flow chart illustrating the enrollment process of study participants and clinical indicators considered for eligibility of genetic testing. TNBC, triple-negative breast cancer; HER2, human epidermal growth factor receptor-2 positive; N/A, not available.

A mixed-methods approach employing both qualitative and quantitative data collection through an embedded sequential exploratory design was employed to report on the process used to obtain informed consent from 97 eligible breast cancer patients (Figure 1) and return of actionable research findings.

### Informed Consent Process

The study population consisted of 97 patients with histologically confirmed breast cancer who attended the Moi Teaching and Referral Hospital (MTRH) for treatment between 2013 and 2016. The ICF used was adopted with permission from a South African genetic study that makes provision for feedback of research results to participants, based on the successful introduction of *BRCA1/2* mutation testing in private practice and at Tygerberg Academic Hospital (Kotze et al., 2004; Schoeman et al., 2013). It was explained to participants that genetic results interpreted in a clinical context may be made known in cases (1) with a definite risk for developing (a second) breast cancer, (2) with a predisposition or risk factor(s) that is treatable or avoidable e.g., by lifestyle or dietary modification, and/or (3) who may need genetic counseling. A material transfer agreement was signed between Moi University in Kenya and Stellenbosch University in South Africa and an import/export permit obtained for sample transport from Kenya to South Africa.

### Data Collection

The consent form was piloted in 16 females who presented with breast lumps, to gauge its ability to clearly and sufficiently relay information regarding the design and goals of the study conducted at MTRH. Where necessary, an interpreter who could speak the patient’s native language was engaged to help translate the information in the consent form. In order to determine whether the patients understood what was discussed during the informed consent process, they were asked to repeat in their own words what had been explained to them. A questionnaire was administered to obtain demographic characteristics, disease status, medication use, family history and lifestyle risk factors as previously described (Busakhala and Torrorey, 2012; Sawe et al., 2016).

Evaluation of the family history was performed using the breast cancer referral screening tool (B-RST^TM^) described by Bellcross et al. (2009). This table was used to record both patient responses to family history questions and to assess eligibility for genetic testing. Two or more checks in the table were regarded as positive, whereas the absence of family history or less than two checks were classified as negative. The selection of two parameters as the cut-off for a positive screen was adopted from published criteria used to define risk associated with highly penetrative, hereditary cancer syndromes (Bellcross et al., 2009).

### Immunohistochemistry

Tumor histopathology was recorded and immunohistochemistry (IHC) of estrogen receptor (ER), progesterone receptor (PR), and human epidermal growth factor receptor-2 (HER2) determined as previously described (Sawe et al., 2016, 2017). Tissue samples were sectioned and fixed onto Flex IHC slides (Dako, Inc.), deparaffinized and hydrated, followed by antigen retrieval using the PT Linker system. The IHC staining was performed using a Dako Cytomation Autostainer Plus, followed by Hematoxylin nuclear counterstaining. For quality control purposes, known positive and negative control specimens were included for each antibody.

### Genetic Studies

DNA was extracted from saliva using Oragene reagents (Ottawa, ON, Canada), according to the instructions provided with this commercially available kit. All DNA preparation steps and analyses were performed at the Pathology Research and Central Analytical Facilities of Stellenbosch University by medical scientists registered with the Health Professions Council of South Africa (HPCSA). Given the lack of a regulatory framework for application of WES in Kenya, only pathogenic *BRCA1/2* variants with well-established clinical guidelines were reported in this study, paving the way for extended WES data analysis and return of research results in Kenya.

Whole exome sequencing (WES) and mapping of sequenced data were performed as previously described by van der Merwe et al. (2017). The Ion AmpliSeq^*rmTM*^ Exome RDY Library Preparation protocol was used and template amplification was performed using the Ion PI^TM^ Template OT2 200 Kit v3 (Thermo Fisher Scientific, Waltham, MA, United States). Semi-conductor sequencing on the Ion Proton system was performed using the Ion PI^TM^ Sequencing 200 Kit v3 with the Ion PI^TM^ Chip Kit v2. This method is designed to target all human exons, the coding regions of the human genome. A coverage depth of 15× was used for detection of potentially causative gene variants. In addition to the quality filters applied, rare variants were filtered on a population frequency of <0.01% with a variant function set to exclude all synonymous variants.

Resulting variant call format (VCF) files were processed using the GeneTalk and wANNOVAR web-based tools for filtering and annotation of sequencing data. Annotated variants were downloaded and filtered for detection of variants in the *BRCA1/2* genes associated with hereditary breast and ovarian cancer using a shell script. The script uses the *grep* command to search in the unfiltered VCF file for variants in the genes on the target gene list, which were sorted with Excel according to variant allele frequency and effect on the relevant amino acids. Bioinformatics tools freely available on the internet (Varsome^1^ and ClinVaR)^2^ were used for variant classification. These automatic variant classifiers evaluate the submitted variant according to the American College of Medical Genetics and Genomics (ACMG) guidelines (Kopanos et al., 2018). Each pathogenic criterion is weighted as very strong (PVS1), strong (PS1–4), moderate (PM1–6), or supporting (PP1–5), and each benign criterion is weighted as stand-alone (BA1), strong (BS1–4), or supporting (BP1–6) (Richards et al., 2015). Rare variants were verified with the Integrative Genomics Viewer, IGV 2.4 and Sanger sequencing used as the gold standard to confirm potential pathogenic variants detected in germline DNA.

### Return of Research Results

Whole exome sequencing reports were generated for return of research results using the PSGT algorithm previously described by van der Merwe et al. (2017). This involves a questionnaire-based assessment and evaluation of tumor molecular subtype as a pre-screen step for eligibility assessment and clinical interpretation of WES. PSGT was applied in this study to help distinguish between inherited breast cancer and patients with lifestyle-related risk factors associated with increased recurrence risk. Relevant data entered in the research database were extracted semi-automatically into adaptable reports using the Gknowmix^TM^ research translation tool^3^ (Kotze et al., 2013). WES reports were authorized for disclosure of actionable results by HPCSA registered medical scientists as part of a multi-disciplinary team responsible for the dissemination of genetic results. The data supporting the conclusions of this manuscript will be made available by the authors, without undue reservation, to any qualified researcher.

## Results

### Informed Consent

One hundred and seven breast cancer patients were invited to participate in this study, of which 10 patients declined to provide consent for research (Figure 1). For assessment of family history across three generations, the B-RST^TM^ family history tool was added to the consent form subsequent to a pilot phase conducted in 16 individuals. Between 30–60 min was spent with each patient to ensure that they understood the purpose of the study and why personal, family and lifestyle information was requested before a saliva sample was collected. The ICF was signed, and a saliva sample collected from 97 breast cancer patients after sufficient comprehension was demonstrated by each individual. Two patients requested that their saliva/DNA samples be destroyed at the completion of the study.

### Clinical Characteristics

Table 1 summarizes the clinical characteristics of the study population, including 2 males and 95 females between the ages of 18 and 80 years, with a mean age of 46.9 years (SD 13.1). Family history of breast/ovarian cancer, male or bilateral breast cancer and triple-negative cancers were considered important clinical indicators for differential diagnosis of inherited and lifestyle-related breast cancer. Family history of cancer was reported in 13 female patients (13.5%, aged 35–70 years). Four patients were below the age of 40 years; one had bilateral cancer; and three breast cancers were triple-negative according to ER, PR, and HER2 status The majority of study participants (62.0%) were diagnosed with cancer before the age of 50 years, with a family history reported in seven (54.0%) of these patients.

**TABLE 1:** Clinical characteristics of 97 Kenyan breast cancer patients included in the study.

Variables	Number	Percentage (%)
Age (years): Mean (SD) 46.9 (13.1)		
Gender		
Female	95	97.9
Male	2	2.1
Pathology		
Adenocarcinoma	1	1.0
Ductal cell carcinoma	23	24.0
Infiltrating ductal carcinoma	35	36.5
Lobular carcinoma	2	2.1
Malignant phyloid tumor	1	1.0
Mucinous	3	3.1
Grade		
I	5	5.2
II	22	22.9
III	17	17.7
IV	4	4.2
Breast cancer type		
Luminal	22	22.6
Triple negative	8	8.2
HER2	4	4.2
Missing	63	65.0
Family History		
No	84	86.6
Yes	13	13.4

### Genetic Results

**FIGURE 2:**
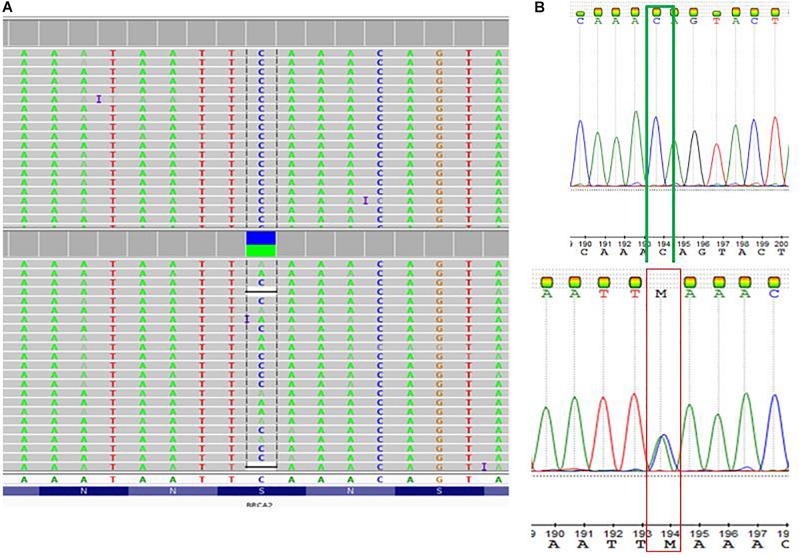
Detection of a pathogenic *BRCA2* variant c.5159C>A; S1720^∗^ (rs80358740; NM_000059.3.1). **(A)** Visualization of whole exome sequencing results using the Integrative Genome Viewer software tool. **(B)** The C to A base change at Genomic location 13: 32339514 (GRCh38) GRCh38 UCSC was confirmed by Sanger sequencing.

Table 2 summarized the tumor type, available IHC data and variants detected in the *BRCA*1 and *BRCA2* genes in 13 patients with familial breast cancer. These include five variants of uncertain significance (VUS) in four patients, and a pathogenic variant c.5159C>A (S1720^∗^) in exon 11 of the *BRCA2* gene. In the corresponding protein of the *BRCA2* nonsense variant, codon 1720 is changed to a stop codon resulting in premature protein truncation. This variant was confirmed by Sanger sequencing (Figure 2).

**TABLE 2:** *BRCA*1/2 gene variants identified in Kenyan breast cancer patients with a family history of cancer evaluated in relation to tumor type and immunohistochemistry used as a proxy for molecular subtype.

CASE	Age	Tumor Pathology	ER	PR	HER2	Laterality	CHR	GENE	NUMBER	REF	ALT	EFFECT	EXON	DNA	PROTEIN	VERDICT	Global MAF
RT053	45	IDC	POS	POS	NEG	BL	17	BRCA1	rs561998108	C	G	Missense	10	c.923G>C	p.S308T	VUS	0.0002
RT053	45	IDC	POS	POS	NEG	L	13	BRCA2	rs80358965	T	G	Missense	15	c.7438T>G	p.L2480V	VUS	0.0004
RT045	52	IDC				L	13	BRCA2	rs74047012	C	T	Missense	15	c.7601C>T	p.A2534V	VUS	0.0006
RT077	35	DCC				L	13	BRCA2	rs1060502421	C	G	Missense	16	c.7676C>G	p.S2559C	VUS	0.0000
RT077	35	DCC				L	13	BRCA2	rs55689095	A	G	Missense	16	c.7712A>G	p.E2571G	VUS	0.0002
RT088	51	IDC	POS	POS	NEG	L	13	BRCA2	rs80358740	C	A	Stopgain	11	c.5159C>A	p.S1720X	Pathogenic	0.0100

*IDC, infiltrating ductal carcinoma; DCC, ductal cell carcinoma; CHR, chromosome; MAF, minor allele frequency; VUS, variant of uncertain clinical significance; POS, positive; NEG, negative.*

### Return of Research Results

The patient with the pathogenic *BRCA2* mutation has been informed of this result by the treating clinician, with the assistance of the extended research team. The insights gained during the qualitative phase of the study involving the informed consent process was incorporated into the patient report used during the feedback process as reflected in Table 3. Return of research results according to the feedback plan in the ICF was facilitated by clinician support, taking patient perceptions and cultural barriers into consideration as well as analytical validation and clinical utility of the genetic findings.

**TABLE 3:** Framework for the return of research results based on the content of the informed consent form (ICF) and information included in the research database of 97 Kenyan breast cancer patients.

Review of Informed consent form	Considerations before return of results	Challenges addressed in report
Eligibility assessment based on signed ICF that makes provision for return of research results.	Histopathology and immunohistochemistry results of breast carcinoma obtained from hospital records.	Maintaining confidentiality during the translation of data into the software program for the generation of an adaptable report.
Clinical relevance of genetic findings and option of genetic counseling a pre-requisite for return of research results.	Analytical validation using Sanger sequencing as the gold standard confirmed the pathogenic *BRCA2* variant detected by WES.	*BRCA2* pathogenic variant in a patient eligible to obtain a report, but the same does not necessarily apply to at-risk family members.
Patient samples collected from 2013 before the requirement for researchers to recontact study participants in the event of variant reclassification that came into effect in 2019.	Variant reclassification is highly unlikely in the case of the pathogenic *BRCA2* mutation identified by WES, while it may become necessary for a VUS in future.	Updated WES report template includes a statement that further studies in an extended sample of breast cancer patients and family screening for the same variant may result in a variant reclassification.
Investigators may be conflicted about returning research results, given the knowledge that genetics cannot fully account for phenotypic variability.	Data on medication use and comorbidities captured in the research database are required for return of WES results relevant to breast cancer treatment.	Pathology-supported genetic testing facilitates evaluation of the clinical characteristics of each patient in relation to inherited-, lifestyle- or therapy-induced risk implications.
Approval for data sharing among genome researchers to gain collective knowledge from return of results and follow-up studies.	Long-term participant engagement allows open communication with researchers aiming to close the gap between expectation and reality.	Availability of research translation resources crucial to cover the costs of validating WES results and contacting the participants for extended testing or report updates.

## Discussion

This study addressed the lack of documentation of real-life experiences on the return of research results in rural African settings, where even basic IHC tumor subtyping was not routinely available to breast cancer patients at the time of recruitment. The finding that ER, PR and HER2 status was available in less than 50% of the study population highlights the potential for chemotherapy overtreatment in patients with early stage breast cancer and a missed opportunity to identify patients with triple-negative breast cancer linked to an increased risk of harboring pathogenic *BRCA1* mutations (Chen et al., 2018). Since 2013, this research has evolved from pilot testing in 16 individuals to collection of saliva samples from 97 breast cancer patients and return of actionable research results to one of 13 cases with familial breast cancer. Whether to return individual research results to study participants remains an area of debate due to complexities associated with the potential reclassification of gene variants that may require a change in clinical management. However, non-disclosure of actionable findings may be unethical given the evolving responsibility of researches to recontact patients after reinterpretation of genomic research results (Bombard et al., 2019; Wong et al., 2019). Use of the PSGT algorithm incorporating the previous knowledge that pathogenic *BRCA2* variants are frequently associated with hormone-positive breast carcinoma increased our confidence level in return of WES-generated research results after confirmation by Sanger sequencing.

While the classification of *BRCA2* c.5159C>A (S1720^∗^) as a pathogenic variant is highly unlikely to change, the finding of VUSs in four Kenyan patients may require recontact of these patients in future in the event of reinterpretation, following family screening, functional studies or extended testing of additional unrelated breast cancer patients (Richards et al., 2015). Pathogenic *BRCA2* variants are associated with an increased risk of other cancers, including ovarian and pancreatic cancer identified as an important consideration for surveillance in the Kenyan patient who was eligible for return of research results (Kopanos et al., 2018). Discussion of the uncertainties and potential for reclassification are therefore crucial to ensure that the outcome of genetic test results and impact on the extended family is understood by the treating clinician and patient.

The qualitative phase of this study was piloted in females referred to MTRH for a lumpectomy, following a breast cancer screening campaign in Western Kenya facilitated by the Academic Model Providing Access to Healthcare (AMPATH) community engagement initiative (Busakhala et al., 2016). Patients with benign breast lumps asked many questions related to a perceived risk that surgery may trigger cancer, with less emphasis on the reason for being approached by the investigator. This preparation phase for genetic analysis highlighted the importance of a well-designed ICF to provide patients with clear and adequate information regarding the study design and goals. The knowledge gained from the pilot phase was applied when obtaining informed consent from the 97 breast cancer patients finally enrolled for the quantitative phase of this study. An effort was made to ensure that participants understood how the study related to their breast cancer diagnosis, recurrence risk, and family members. Most of the breast cancer patients needed further clarification of medical terms used in the consent form, such as DNA and hormone replacement therapy. As previously highlighted by de Vries et al. (2015), these terms have no equivalent translation in the native African languages and therefore required extra time for an explanation in an attempt to improve comprehension.

A young patient hesitated to provide consent, but when probed as to why she was not willing to sign the form, she answered as follows; “*I cannot read or write and I am ashamed of people to know about it.”* It has been reported that a major barrier to effective consenting is the level of literacy (Silverman et al., 2005). Although illiteracy is expected to be higher among elder people, it is now evident that even among the young in Kenya, it may still be a problem. Most illiterate individuals are ashamed of this fact and therefore refuse to accept or alternatively ask their next of kin to sign on their behalf. Considering that true informed consent is achieved only if the potential participant understands the purpose, methods, risks, and alternatives of a study in relation to his or her personal clinical context (Emanuel, 2000; Boga et al., 2011; Gupta et al., 2012; Tindana et al., 2012), illiteracy is likely to hinder this process. Similar to other studies, we used an interpreter familiar with the patient’s dialect (Abeybsekera, 2018). A recommendation is that investigators in Kenya and other African countries embrace the utilization of tools that may be of value in low-literacy communities such as the use of picture files or speaking books. The latter resource employs cartoons in addition to text, which sounds when the corresponding button is pushed for a specific concept page. An ongoing pilot on the utility of these speaking books is expected to improve communication and may be available for widespread distribution and use across Africa^4^.

Muthoni and Miller (2010) reported that a diagnosis of breast cancer may lead to a state of low self-esteem, a feeling that may predispose to stigma and trauma and hence could deter both male and female patients from seeking early healthcare advice. Moreover, some patients perceive a diagnosis of breast cancer as a death sentence and may delay treatment in order to protect their families from stigmatization. Rayne et al. (2016) reported a significant relationship between younger age of onset and fear of loss of hair or breasts in an urban population in South Africa. This may also be the case in Kenya, as ten patients who were approached in this study declined signing the ICF. Of these ten patients, one male displayed a “silent refusal attitude” at the beginning, which was characterized by hesitation to participate without openly refusing. Such behavior has previously been reported in a Kenyan study, which did not involve genetic investigations (Kamuya et al., 2015). Reasons for this attitude may be that participants do not want to offend the interviewer, especially after he/she spent their energy and time explaining about the study goals and expected outcomes in a manner that should clarify the difference between routine cancer treatment and potential feedback on genomic research results. Participants may avoid declining the request for voluntary research participation openly when the researcher is seen as part of the hospital setting to ensure continued benefit from the institution (Kamuya et al., 2014). Management of patient fears and expectations following breast cancer diagnosis may curb beneficial misconception in potential participants, while simultaneously ensuring true comprehension and voluntary participation in the research process. This is an important consideration given the reaction of a 20-year old female invited into the study, who ran away from the hospital ward due to fear of surgery. In South Africa, fear of genetic discrimination was identified as the major concern during the implementation of a *BRCA1/2* screening program more than 10 years ago (Kotze et al., 2004). This issue was addressed by weighing the benefits and risks associated with different types of genetic tests and correcting misconceptions in the context of breast cancer, where prophylactic surgery is an option. Knowledge about the role of reconstructive breast surgery may ease the fear in breast cancer patients and could guide risk reduction interventions accessible to Kenyan breast cancer patients.

As advocated by Akuoko et al. (2017), earlier presentation to the hospital could be achieved if healthcare providers, with support from the government, collaborate in developing approaches to improve the clinical outcome of patients with breast cancer. Disparities in genetic testing services available in high versus moderate/low-income countries such as Kenya (Hill et al., 2015), highlighted the need for financial assistance programs to remove economic barriers to breast cancer genetic testing (Jones et al., 2017). A systematic review of the cost-effectiveness of healthcare programs involving genetic testing of the two major breast cancer genes, *BRCA1* and *BRCA2* indicated that family history-based screening programs are cost-effective (D’Andrea et al., 2016). Use of data integration tools such as PSGT incorporating WES to identify individuals at increased risk of inherited versus lifestyle-related forms of cancer or treatment-related co-morbidities, may further improve the cost-effectiveness of testing.

More extended data analysis of WES reads beyond *BRCA1/2* is an important consideration in genetically uncharacterized patients, especially those experiencing medication side effects or treatment failure. The potential value of simultaneous detection of pharmacogenetic markers relevant to tamoxifen resistance (Baatjes et al., 2017) has been mentioned in the feedback report of the pathogenic *BRCA2* variant in one of the Kenyan patients studied. Results from such studies may inform and guide government policy toward the provision of medical coverage for genetic tests to improve patient outcome. The emerging duty to return genetic information is based on the principles of autonomy, beneficence, and the acknowledgment that translational genomic research cannot progress without the engagement of research participants (Lolkema et al., 2013; Bombard et al., 2019). Lack of genetic counselors in Kenya necessitates the development of an innovative approach toward breast cancer genetic testing and return of research results. Incorporating cognitive strategies through a multimedia approach may in future enhance comprehension of research procedures, instead of using a single face-to-face method when consenting and counseling patients in biomedical research (Antal et al., 2017).

**FIGURE 3:**
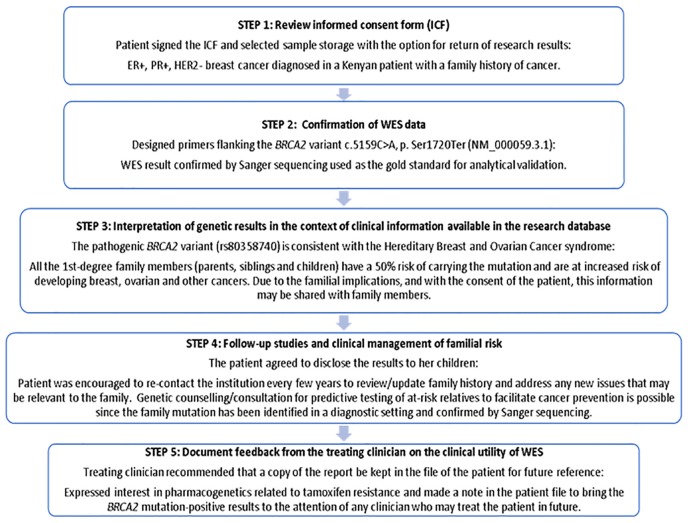
Five-step process used to disclose actionable research results to a study participant with *BRCA*2 pathogenic variant and documenting feedback from the treating clinician to determine clinical utility.

The ICF used in this study clearly indicated that research results may be reported based on clinical relevance and/or the need for genetic counseling that may include support to at-risk family members of study participants. In this context, different models ranging from specific to tiered and broad consent (Munung et al., 2016) were evaluated by Van Der Merwe (2018), who conducted a qualitative study exploring South African stakeholder views on the return of individual research results and incidental findings. Stakeholders including clinicians, genetic counselors and medical scientists contended on the one extreme that broad consent would enable more information to be gathered for future use in research, and that the broad categories of potential harm, potential benefit and limitations should be covered - not necessarily all the details in-between. On the other extreme, some stakeholders advocated for use of tiered consent that allows participants to break down the various aspects of consenting as far as they wish. Explanation of the return-of-results plan to every potential participant prior to study enrollment facilitated disclosure of actionable *BRCA2* results in this study, which may in future be extended to pharmacogenetic WES data analysis using a clinical pipeline applicable to PSGT tailored to the needs of the individual in an adaptable report (Baatjes et al., 2017). The WES report that was generated for the Kenyan breast cancer patient with a pathogenic *BRCA2* variant was used by the treating clinician to disclose the results based on the steps stipulated in Figure 3. Considering that there is no standardized method for returning research results in Kenya, we applied the Clinical Sequencing Exploratory Research (CSER) Consortium and the Electronic Medical Records and Genomics (eMERGE) Network guidelines (Jarvik et al., 2014) based on the ACA (Analytical validity; Clinical significance and Actionability) criteria (Knoppers et al., 2015).

During result disclosure the treating clinician used a language that the patient is comfortable with and informed her about the risks and benefits of *BRCA1/2* gene screening, as explained in the WES report. After gauging her feelings on receiving the genetic results and assessment of the level of understanding before proceeding to deliver the result, the patient confirmed that she still wants to know the outcome (test voluntariness) (Isles, 2013). Our experience in overcoming barriers to the disclosure process due to lack of standardized protocols or consensus guidelines on how and when to return genetic test results, contributed to the development of a framework for tiered informed consent applicable to research in Africa (Nembaware et al., 2019). Although there is a lack of genetic counselors in Kenya, this did not preclude disclosure of the genetic results. Effective feedback was achieved through joint consultation with the researcher who obtained the initial informed consent from the patient and clinicians involved in the genomic research project and treatment of the patient. Since adhering to the gold standard of involving genetic counselors or medical geneticists may not be practical in a resource-limited setting, primary care physicians and primary care nurses need to be empowered by education in this context (Van Der Merwe, 2018).

The results presented in this study are supported by many strengths, such as the inclusion of a qualitative research component that prepared us for potential problems during information transfer in the subsequent study. The initial reactions of Kenyan patients highlighted the needs of the study population during implementation of a genetic screening program, which in turn guided the way in which we approached the informed consent process and disclosure of genetic results. The cultural level and degree of diversity among patients were not assessed and could have affected their reactions and understanding of the goals of the project and hence acceptance to participate in the study.

## Conclusion

This study describes the use of WES to screen for pathogenic *BRCA1/2* variants alongside clinico-pathological assessments used for interpretation of the findings obtained in Kenyan breast cancer patients. The challenges encountered during the informed consent process were contextualized into a framework for return of research results to patients with familial breast cancer. The pre-test process pioneered during this investigation identified problem areas addressed by the development of an adaptable report that enables disclosure of WES results in five steps. Detection of a pathogenic *BRCA2* mutation (c.5159C>A; S1720^∗^), in 1/13 (8%) of the breast cancer patients with familial breast cancer confirmed that family history is an important indicator for the diagnosis of inherited breast cancer in Kenya. Novel insights gained as a result of this experience support the incorporation of new technologies integrated with standard pathology to facilitate the return of research results in low and middle-income settings, where challenges associated with the translation of sophisticated genetic terms into native African languages persist. As genetic research gains momentum on the African continent, innovative strategies such as PSGT combining pathology and genetic tests will become increasingly important to translate research into clinical practice. This is the first study performed in Kenya to determine the cause of familial breast cancer in patients using WES, which allows for extended data analysis beyond *BRCA1/2* at no additional cost in patients who experience treatment failure or medication side effects.

## Disclosure

MK is a director and shareholder of Gknowmix (Pty) Ltd., that developed a database tool for research translation under the auspices of the South African Medical Research Council.

## Data Availability Statement

The raw data supporting the conclusions of this article will be made available by the authors, without undue reservation, to any qualified researcher.

## Ethics Statement

The studies involving human participants were reviewed and approved by the Institutional Review and Ethics Committee (IREC). Moi Teaching and Referral Hospital, Eldoret, Kenya. The patients/participants provided their written informed consent to participate in this study.

## Author Contributions

RT-S, SM, and MK made substantial contributions to the conception and design of this project. RT-S obtained informed consent and clinical data. RT-S and MK wrote the manuscript. NM gave a genetic counseling perspective. SM and NM critically revised the manuscript. All authors read and approved the final manuscript.

## Conflict of Interest

The authors declare that the research was conducted in the absence of any commercial or financial relationships that could be construed as a potential conflict of interest.

## Footnotes

1 https://varsome.com

2 https://www.ncbi.nlm.nih.gov/clinvar

3 https://www.gknowmix.org

4 http://www.booksofhope.com/, n.d.
